# Probiotic and Rice-Derived Compound Combination Mitigates Colitis Severity

**DOI:** 10.3390/ph17111463

**Published:** 2024-10-31

**Authors:** Ashraf Khalifa, Mayyadah Abdullah Alkuwayti, Basem M. Abdallah, Enas M. Ali, Hairul Islam M. Ibrahim

**Affiliations:** 1 Biological Science Department, College of Science, King Faisal University, P.O. Box 400, Al-Ahsa 31982, Saudi Arabia; 2 Botany and Microbiology Department, Faculty of Science, Beni-Suef University, Beni-Suef 62511, Egypt; 3 Molecular Biology Division, Pondicherry Centre for Biological Sciences and Educational Trust, Pondicherry 605004, India

**Keywords:** *Enterococcus lactis*, Al-Asfar Lake, inflammatory disorders, Hasawi rice, protein hydrolysate

## Abstract

Background: This study investigated the ability of *Enterococcus lactis* (*E. lactis*) and Hasawi rice protein lysate (HPL) to suppress colitis induced by dextran sulfate sodium (DSS) in miceColitis is characterized by inflammation of the colon, and exploring potential therapeutic agents could lead to improved management strategies. Methods: Male mice were subjected to DSS treatment to induce colitis, followed by supplementation with *E. lactis* and/or HPL. The study assessed various parameters, including disease activity index (DAI) scores, gut permeability measured using FITC-dextran, and superoxide dismutase (SOD) activity in excised colon tissues from both treated and untreated control groups. Results: *E. lactis* supplementation significantly alleviated DSS-induced colitis, as evidenced by improved DAI scores and enhanced gut permeability. Notably, *E. lactis* combined with HPL (0.1 mg/10^8^) exhibited superior tolerance to a 0.5% pancreatin solution compared to *E. lactis* alone. Both *E. lactis* and the combination treatment significantly increased SOD activity (5.6 ± 0.23 SOD U/mg protein for *E. lactis* and 6.7 ± 0.23 SOD U/mg protein for the combination) relative to the Azoxymethane (AOM)/DSS group, suggesting a reduction in oxidative stress. Additionally, pro-inflammatory markers were significantly reduced in the group receiving both *E. lactis* and HPL compared to the E. lactis-only group. Levels of proteins associated with cell death, such as PCNA, PTEN, VEGF, COX-2, and STAT-3, were significantly decreased by 14.8% to 80% following *E. lactis* supplementation, with the combination treatment showing the most pronounced effects. Conclusions: These findings suggest *E. lactis* supplementation may be beneficial for colitis, with HPL potential to enhance its effectiveness.

## 1. Introduction

Colorectal cancer (CRC) represents a significant global health concern, ranking as the third most prevalent cancer and the second leading cause of cancer-related mortality worldwide [1]. CRC originates from the epithelial cells lining the colon or rectum, and its pathogenesis is characterized by significant complexity. Metastasis, a critical condition associated with CRC, often contributes to the negative outcomes observed in most CRC patients [2]. The etiology of CRC includes a range of factors, such as dietary habits, lifestyle choices, and hereditary predispositions that promote abnormal cellular proliferation and an increased likelihood of invasion and metastasis. Chronic infection has been linked to carcinogenesis, with an increased risk of CRC associated with the duration and severity of colitis, as well as the presence of various inflammatory conditions [3]. The interplay between these factors and gut microbiota plays a crucial role in CRC by affecting metabolism, barrier integrity, and immune regulation [4,5]. Anti-inflammatory drug treatment inhibits the progression of IBD (inflammatory bowel disease) to CRC [6]. However, these treatments also bring up serious side effects [7]. Therefore, in an attempt to identify a more effective and safer strategy to treat IBD and avert its progression to CRC, synbiotics have been identified. Synbiotics, compromising both prebiotics and probiotics, can modulate gut microbiota, promoting a healthy microbiome. Probiotics are beneficial bacteria that improve host health, encompassing genera such as *Bifidobacterium*, *Lactobacillus*, and *Streptococcus*. Prebiotics are indigestible dietary components that significantly stimulate the growth and activity of beneficial microbiota in the host gut. Therefore, they exert positive effects on the host, such as inulin, fructooligosaccharides (FOS), and galactooligosaccharides [8,9].

Probiotics have garnered interest due to their anti-inflammatory, antioxidant, and intestinal microbial regulatory activity [5,10]. Previous research identified *E. lactis* isolated from Al-Asfar Lake, Saudi Arabia, as displaying probiotic properties, demonstrating resilience to stressors, gastric transit, and harsh circumstances [10,11]. The mechanisms underlying the observed effect of *E. lactis* (ASF-2) may also involve the release of bioactive compounds that can inhibit the production of anti-inflammatory enzymes, scavenge unfastened radicals, or modulate the production of inflammatory cytokines [11]. *E. lactis* traces are known for their therapeutic potential, as they can change the host’s microbiota, immune response, and metabolism by making antimicrobial receptors and controlling the immune system. This aligns with recent research highlighting the multifaceted advantages of probiotics in numerous disease fields. For instance, Khalifa et al. [12] investigated the effects of *Bacillus amyloliquefaciens*-rich camel milk on mice with colitis, indicating its potential as a therapeutic agent for IBD. Khalifa and colleagues’ research demonstrated that probiotic-fermented camel milk mitigated neurodegenerative signs in mouse models by regulating the SOX5/miR-218 axis. The results indicated that probiotics possess neuroprotective properties. In addition, Ibrahim et al. [13] found that the addition of *B. amyloliquifaciens* to camel milk reduced neuroinflammation in a mouse model of autoimmune encephalomyelitis by controlling inflammatory markers such as IL-1β, IL-6, IL-17, and TNF-α. Furthermore, Khalifa and Islam [5] illustrated that *Enterococcus faecium* isolated from bird feces advanced hen immune response and alleviated Salmonella infections, suggesting the role of probiotics in improving host immunity and preventing pathogen colonization. Khalifa et al. [10] found that the probiotic bacterium *Lactococcus* sp. PO3 attenuated immunogenicity in myelin oligodendrocyte glycoprotein-induced oligodendrocytes, suggesting its potential to alleviate autoimmune responses. Overall, these findings highlight the diverse therapeutic potential of probiotics in influencing immune characteristics and alleviating inflammatory and autoimmune disorders, as well as the significance of probiotic research in clarifying their mechanisms of action and clinical applications.

Despite its nutritional richness, Hasawi rice from the eastern region cultivars of Saudi Arabia was selected for this study. The Al-Ahsa Oasis’ local cuisine is characterized by this resilient rice variety, which is highly regarded for its capacity to withstand harsh conditions. This rice showcases the enduring traditions and adaptability of Saudi Arabia’s agricultural heritage. The geological landscape in an arid climate with scorching temperatures of up to 48 °C/118 °F [14]. The use of HPL in CRC mitigation remains underexplored. Hasawi rice contains nutrients, minerals, fibers, proteins, and unsaturated fats. The rice proteins comprise albumin, globulin, glutelin, and prolamin [15,16]. Among these proteins, albumin exhibits the highest phytate content in rice bran, while prolamin demonstrates the lowest content in Hasawi rice. Rice albumin offers potential as a source of protein hydrolysates, undergoing enzymatic hydrolysis with alcalase enzymes, which have shown effectiveness in glutelin degradation. The albumin content constitutes the primary rice protein, accounting for 60–80% of total protein content [17]. Studies suggest that Hasawi rice protein hydrolysate (HPL) has beneficial effects on intestinal fitness, as well as the inhibition of infection and associated barrier disorders in colitis models that are precipitated by the use of dextran sulfate sodium (DSS) and acetic acid in animal models [12,18].

While researchers have elucidated the physiological benefits of rice protein and *E. lactis*, their combined synergistic effects on antitumor activity and intestinal health, including immune regulation and intestinal microbiota stability, are still largely unexplored [15,16,17]. Previous models of anti-inflammatory interest have affirmed the inhibitory impact of *E. lactis* in mouse models [11]. Therefore, this study aims to assess the potential synergistic effects of *E. lactis* and HPL prebiotics in the restoration and treatment of colon damage caused by AOM/DSS in C57BL/6j mice.

## 2. Results

### Assessment of the Survival of Probiotics in the Gastrointestinal Tract by Pancreatin Tolerance Testing

**Figure 1:**
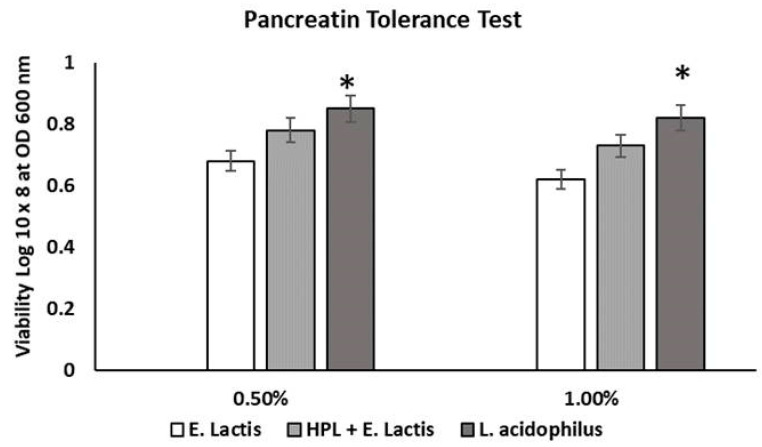
A pancreatin tolerance test involved the addition of 100 µL of *E. lactis* and *L. acidophilus* (ATCC strain 4356) to MRS broth containing 0.5% pancreatic. The mixture was subsequently incubated at 37 °C for a duration of 24 h. Optical density (OD) was assessed at a wavelength of 600 nm. In the experiments, Hasawi protein lysate (HPL) was utilized at a concentration of 0.1 mg per 10^8^ CFU culture load. *E. lactis* was present at a concentration of 1 × 10^8^ CFU/mL, HPL contained *E. lactis* at the same concentration, and *L. acidophilus* was cultured in MRS broth supplemented with 0.5% pancreatin. Each experiment was conducted independently a minimum of three times (n = 3 biological replicates per group). Data are expressed as mean ± standard deviation (SD). Statistical significance was assessed by comparison with the free probiotic group (* *p* < 0.05).

The ability of probiotics to survive in the upper gastrointestinal tract and tolerate pancreatic juice indicates their probiotic properties. The growth kinetics of test probiotic isolates were determined by measuring the absorbance after 24 h of incubation (Figure 1). *E. lactis* combined with (0.1 mg/10^8^ CFU *E. lactis*) demonstrated superior tolerance to a 0.5% pancreatin solution compared to *E. lactis* alone. The HPL group exhibited a tolerance of 0.721 ± 0.05 × 10^8^, whereas the *E. lactis* group demonstrated a tolerance of 0.67 ± 0.04 × 10^8^. These results were significantly similar to *L. acidophilus* strains (0.82 ± 0.04 × 10^8^) (Figure 1).

**Figure 2:**
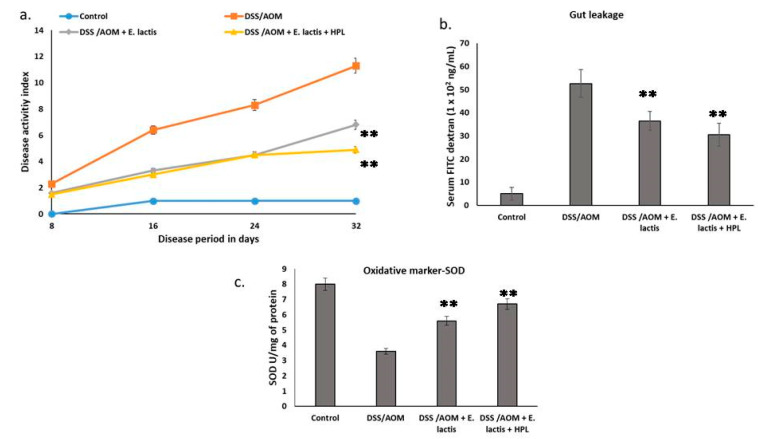
Effect of *E. lactis* oral administration on the DAI of the AOM/DSS-treated C57BL/6J mice (n = 6). The mice were divided into two groups: Group 1 served as the control group, and Group 2 was the treatment group. Mice received a single intraperitoneal (i.p.) injection of AOM (10 mg/kg) and subsequently received 2.5% DSS in their drinking water for one week, followed by two weeks of regular drinking water for recovery; this treatment cycle was repeated three times. Group 3 received DSS/AOM with *E. lactis* 10^8^), while Group 4 received DSS/AOM with *E. lactis* and HPL at a dosage of 1 mg/100 g body weight. (**a**) DAI was scored for DSS/AOM mice with a 32-day disease period. (**b**) The study displays the gut leakage (FITC-dextran assay) in a dextran sulfate solution (DSS)-induced colitis mouse model, a representative IBD model. Mice body weight; (**c**) SOD activity was measured in units per milligram of protein (U/mg protein). The data present the mean ± standard deviation (SD). Asterisks denote the significance of *E. lactis* and HPL + *E. lactis* versus the AOM/DSS group, as determined by one-way ANOVA (** *p* < 0.01).

We studied the effects of *E. lactis* on DAI scores, gut leakage, and antioxidant stress in mice with DSS-induced colitis. Figure 2 demonstrates the potential therapeutic benefits of *E. lactis* supplementation in mice (with and without HPL) at a dosage of 1 mg/kg body weight in alleviating AOM/DSS-induced colitis. The AOM/DSS mice exhibited severe colitis, indicated by heme leakage in the feces, a sign of inflammation in the intestines (Figure 2a–c). This resulted in weight loss and bloody diarrhea, as shown in Figure 2a. The consolidated disease index observation also included the crypt foci of colon and tumor colonization.

Interestingly, the DAI score of the *E. lactis* and HPL (HPL + *E. lactis*) group showed a significant recovery compared to the group receiving only *E. lactis*. This finding indicates the synergistic effect of *E. lactis* and HPL on enhancing gut barrier integrity. The HPL + *E. lactis* group exhibited reduced gut leakage, as indicated by lower FITC-dextran levels, compared to the *E. lactis* group (Figure 2b).

**Figure 3:**
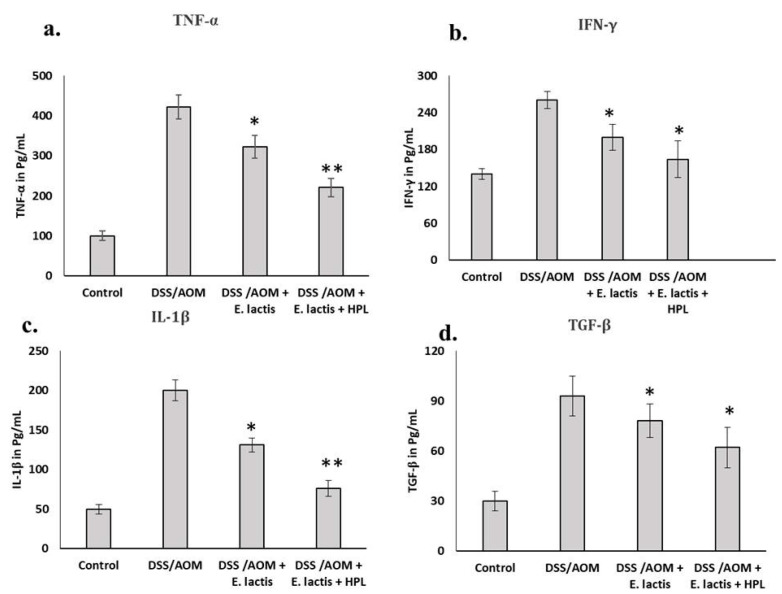
*E. lactis* inhibits DSS-induced inflammation in the colon of mice. Colon tissue pro-inflammatory cytokine (**a**) TNF-α, (**b**) IFN-γ, (**c**) IL-1β, and (**d**) TGF-β contents, detected by ELISA. The treatment conditions include DSS/AOM with and without *E. lactis,* as well as DSS/AOM with *E. lactis* in conjunction with HPL. Data for all quantifications (mean ± SD) were derived from the specified number of independent experiments and analyzed using a two-way ANOVA (* *p* < 0.05 vs. the control group and the DSS/AOM-induced colitis-associated group; ** *p* < 0.01 vs. the control group and the DSS/AOM-induced colitis-associated group).

Additionally, the effect of *E. lactis* supplementation, with and without HPL, on SOD activity in the colons of mice with AOM/DSS-induced colitis (Figure 2c) was investigated. The mice in the non-colitis group demonstrated the highest SOD activity (7.6 ± 0.33 SOD U/mg protein), indicating their potent antioxidant defense system. In contrast, mice with AOM/DSS-induced colitis exhibited significantly lower SOD activity (3.6 ± 0.41 SOD/mg protein). The administration of *E. lactis* alone or combined with HPL significantly increased SOD activity compared to the AOM/DSS group (5.6 ± 0.23 SOD U/mg protein and 6.7 ± 0.23 SOD U/mg protein, respectively). The effects of *E. lactis* supplementation, with or without HPL, on inflammatory markers in mice with AOM/DSS-induced colitis are shown in Figure 3.

**Figure 4:**
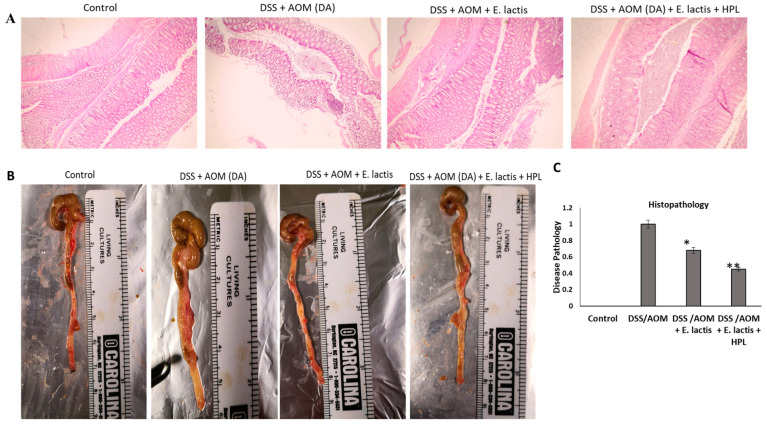
*E. lactis* effect on DSS-induced colitis symptoms in a mouse model. The treatment conditions include DSS/AOM with and without *E. lactis*, as well as DSS/AOM with E. lactis combined with HPL. On day 32 of the experiment, a representative photograph and statistical analysis of colon length were conducted. H&E staining of representative histological sections of colons from the groups (200× magnification). (**A**) The colon and histopathology scores show representative changes in histology. Low Mag: Scale bars, 20 μm. (**B**) The macroscopic examination of DSS/AOM with and without *E. lactis* and DSS/AOM with *E. lactis* with HPL colon. (**C**) The histological score was calculated as described in Methods. H&E staining was performed. For all quantifications, the data (mean ± SD) were from the indicated number of independent experiments and analyzed using a one-way ANOVA (* *p* < 0.05 vs. the DSS/AOM disease group; vs. the DSS-induced colitis group * *p* < 0.05 vs. ** *p* < 0.01).

This study demonstrated that mice administered *E. lactis* (with or without HPL) exhibited significantly reduced levels of all four pro-inflammatory markers compared to the AOM/DSS group. The levels of TNF-α, IFN-γ, IL-1β, and TGF-β were substantially lower in these mice. The reduction in pro-inflammatory markers was significantly higher in the group receiving both *E. lactis* and HPL than in the group receiving only *E. lactis*. Figure 3 illustrates that the levels of inflammatory markers TNF-α (322 ± 28 pg/mL), (221 ± 23 pg/mL), IFN-γ (200 ± 21 pg/mL), (164 ± 30 pg/mL), (c) IL-1β (131 ± 9 pg/mL), (76 ± 10 pg/mL), and (d) TGF-β (78 ± 10 pg/mL), (62 ± 12 pg/mL), showed a significant negative expression by AOM/DSS with *E. lactis* with and without HPL treatment compared to the AOM/DSS-induced colitis-induced groups (422 ± 30 pg/mL), (260 ± 14 pg/mL), (200 ± 13), pg/mL) (>0.042), and (93 ± 12 pg/mL) (Figure 3a–d). These findings indicate that *E. lactis* with HPL can reduce AOM/DSS-induced CRC by regulating inflammatory cytokines levels and potentially inhibiting TGF-β secretion from 91 pg/mL to 59.3 pg/mL. The results indicated that the administration of *E. lactis* combined with HPL had a more significant impact on inhibiting AOM/DSS CRC compared to the administration of *E. lactis* alone. The histopathological examination of colon sections from mice revealed inflammation and immune cell infiltration in the inflamed tissue regions, observed at crypt foci and cell masses in select areas of the distal colon (Figure 4A).

The histopathological examination of the colon sections from mice revealed inflammation and infiltration of immune cells in the inflamed tissue regions and found crypt foci and cell masses in a few areas of the distal colon (Figure 4A).

**Figure 5:**
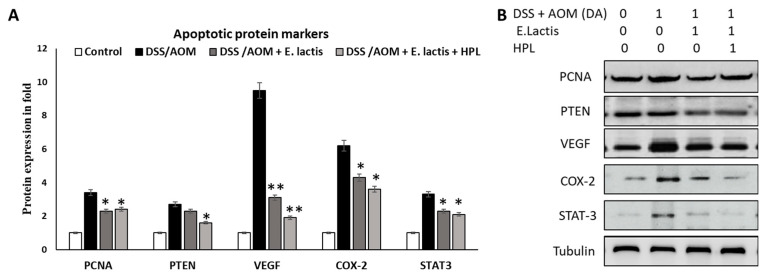
*E. lactis* reduces the expression of apoptotic protein markers in the colons of mice with DSS-induced colitis. The treatment conditions include DSS/AOM with and without *E. lactis*, as well as DSS/AOM with *E. lactis* in combination with HPL. Colon tissues were excised following a duration of 35 days of disease. (**A**,**B**) Analysis of PCNA, PTEN, VEGF, COX-2, and STAT-3 protein marker levels in the colon, along with quantification data. Data for all quantifications (mean ± SD) were derived from the indicated number of independent experiments and analyzed with a one-way ANOVA. * *p* < 0.05, compared to the DSS/AOM disease group (* *p* < 0.05, ** *p* < 0.01).

The macroscopic examination revealed that the colon length was increased by 5.7 cm to 7.6 and 8.2 cm in *E. lactis* and *E. lactis* with HPL, respectively; Figure 4B. The correlation with the slice indicated a significant reduction in cellular proliferation (*p* < 0.01) in both E. lactis and DSS/AOM groups treated with *E. lactis* in HPL mice. The findings indicate that *E. lactis,* in combination with DSS/AOM and HPL, alleviated the adverse effects of AOM/DSS on cell proliferation in colitis. Supplementation with *E. lactis*, with or without HPL, significantly alleviated disease pathology in DSS-induced colitis mice. The effects of *E. lactis* supplementation (with and without HPL) on apoptotic protein markers in mice with AOM/DSS-induced colitis are illustrated (Figure 5). Mice with AOM/DSS colitis exhibited significantly elevated levels of proteins associated with cell death, including PCNA, PTEN, VEGF, COX-2, and STAT-3. Supplementation with *E. lactis* (with or without HPL) resulted in a significant reduction in these proteins by 14.8% to 80% in comparison to the colitis group. The group that administered both *E. lactis* combined with HPL exhibited a more substantial reduction, indicating that HPL may augment the protective effects of *E. lactis*.

Mice that received *E. lactis* supplementation (with or without HPL) demonstrated significantly reduced expression of all four apoptotic protein markers by 14.8% to 67.3% and 29.4 to 80%, respectively, compared to the AOM/DSS group, which had higher levels of PCNA (3.4-fold), PTEN (2.7-fold), VEGF (9.5-fold), COX-2 (6.2-fold), and STAT-3 (3.3-fold) (Figure 3a–d). The group that administered both *E. lactis* and HPL exhibited a significantly more significant reduction in inflammatory markers than the group that received only *E. lactis*.

Furthermore, the group that administered both *E. lactis* and HPL demonstrated a significant reduction in pro-inflammatory markers, suggesting that HPL may augment the protective effects of *E. lactis.*

## 3. Discussion

Probiotics have demonstrated efficacy in reducing gut inflammation and regulating cytokine levels, indicating their potential as a therapeutic option for colitis-associated colorectal cancer [2,3]. Research on probiotics has highlighted their health benefits, particularly in colorectal cancer related to colitis, with promising findings indicating a positive impact on gut microbiota. This encompasses improvements in functionality, decreases in inflammation, and the suppression of the proliferation of cells associated with colorectal cancer [19]. The capacity of intestinal lactobacilli to endure adverse conditions is essential for assessing their characteristics, as indicated by the Food and Agriculture Organization of the United Nations (FAO/WHO) [20] and Wells & Mercenier et al. [21]. The present study assessed the survival of bacterial strains in the gut. The findings revealed that pancreatin at concentrations of 0.5% and 1.0% weight-per-volume did not eradicate strains of *E. lactis*, *E. lactis* HPL, and *L. acidophilus* ATCC after 24 h of incubation. The results indicate that HPL may enhance probiotic activity. However, an extended incubation time resulted in a decrease in the viability count of *E. lactis*.

A substantial body of evidence supports the health benefits of the host’s gut microbiota, particularly by identifying beneficial bacteria from diverse sources that can reduce inflammatory cytokines across different hosts [5,10,12]. The findings of the current study align with those of previous reports [5,15]. Probiotics have potential health benefits, provided they can survive transit through the upper gastrointestinal tract and tolerate pancreatic juice within the host system. Pancreatin tolerance assesses the ability of probiotic microorganisms to withstand the effects of digestive enzymes.

Hasawi rice is a significant source of dietary fiber, minerals, vitamins, and phytochemicals. Rice bran, a by-product of rice processing, has garnered interest due to its bioactive properties. Phenolic extracts derived from rice bran have demonstrated antioxidant and anti-inflammatory effects, protecting against dysfunction in human primary cells [22]. In randomized studies, ferulic acid esters and phytosterols extracted from rice bran have improved antioxidant status in hyperlipidemic subjects [23,24]. The current study demonstrates that a protein extract from Hasawi rice markedly decreased colonic levels of IL-6 and T-helper-cell-associated cytokines, including TNF-α, while reversing TGF-β secretion in mucosal colon tissues. Recent studies indicate that alcohol extracts of rice bran significantly enhance claudin-4 protein expression and reduce UC-elevated GSH levels in the colon [25,26].

Furthermore, Elkholy et al. [17] demonstrated that probiotics have the potential to enhance the integrity of the colonic epithelial barrier and reduce oxidative stress, both of which are crucial for the treatment and prevention of CRC associated with severe colitis. Pretzsch et al. [2] propose the use of protein hydrolysates derived from Hasawi rice to investigate the potential synergistic effects of probiotics and prebiotics, which may improve their combined impact on gut health. This combination may offer a more comprehensive strategy for improving colon health and reducing the risk of cancer diagnosis. Additionally, the anti-inflammatory properties of probiotics offer a significant rationale for their efficacy in preventing or treating colitis-associated CRC [17]. Probiotics may reduce chronic inflammation linked to colitis by modulating gut microbiota and cytokine markers, potentially averting the progression of colitis to CRC [27]. However, further research is necessary to elucidate the precise mechanisms underlying the effectiveness of this therapeutic approach. This study investigated the efficacy of *E. lactis*, with and without HPL, in reducing colitis in mice induced by AOM/DSS. The AOM/DSS group exhibited significant gut inflammation, characterized by heme leakage in feces, weight loss, and the presence of bloody diarrhea (see Figure 2a). The observed symptoms suggest that the intestinal barrier has been compromised, allowing blood components to enter the feces. The group receiving both *E. lactis* and HPL showed more improved gut health than the *E. lactis* group. These results are similar to those from earlier studies that showed *L. rhamnosus* LM07 and *L. plantarum* LM17 improved colitis symptoms in a DNBS-induced mouse model by lowering intestinal permeability, as shown by lower levels of FITC-dextran in the serum and myeloperoxidase (MPO) in colonic tissue [27,28]. Recent studies have also identified the secreted protein HM0539 from LGG as characterized by protective effects on the intestinal barrier.

This suggests a potential synergy between *E. lactis*, which affects gut microbiota, and HPL, strengthening the intestinal barrier. Therefore, *E. lactis* supplementation, particularly with HPL, may effectively treat colitis by strengthening the gut barrier and reducing inflammation. Colic-SOD activity significantly decreased in AOM/DSS-induced colitis models [28], suggesting a compromised antioxidant defense. Probiotics may enhance oxidative stress regulation. For instance, *L. fermentum* expresses manganese superoxide dismutase (Mn-SOD) [29,30], which can decrease mitochondrial reactive oxygen species (ROS). Probiotics also modulate antioxidant signaling pathways by stimulating the phosphorylation of antioxidant enzyme transcripts [31].

The addition of *E. lactis*, with or without HPL, led to a significant increase in SOD activity compared to the colitis group. This result suggests that *E. lactis* may have anti-inflammatory effects or change the microbiota in a way that boosts the body’s natural antioxidant response. Further research is necessary to evaluate the significance of HPL co-treatment and to explore potential synergistic mechanisms with *E. lactis.* Although HPL co-treatment resulted in a slight increase in SOD activity, this finding does not validate its efficacy.

Pro-inflammatory cytokines released from damaged intestinal mucosa serve as critical markers of immune cell hyperactivation subsequent to the induction of IBD [32]. Damage-induced immune responses elevate cytokine levels, including TNF-α, IL-1β, and IFN-γ. Dysregulated IL-6 synthesis can negatively affect autoimmunity and disrupt TGF-β regulation, which protects against colitis [33]. This study demonstrated that the combination of HPL and *E. lactis* resulted in a reduction in IL-6, IL-1β, and TNF-α levels while simultaneously increasing TGF-β levels. The results indicate that the oral administration of *E. lactis* combined with HPL reduces colonic damage, which was not observed in untreated mice.

Moreover, significant alleviation of oxidative stress was indicated by decreased malondialdehyde (MDA) levels and increased SOD activity. Both rice protein and *E. lactis* affected signaling pathways involving PCNA, PTEN, VEGF, COX-2, and STAT-3. These results support a synergistic approach for the simultaneous use of probiotics and Hasawi rice protein in the treatment of ulcerative colitis.

Excessive cytokine production and ROS activate various transcription factors that stimulate inflammatory responses. Inflamed colonic cells express significant levels of proliferative and inflammatory mediators, including PCNA [12]. This study demonstrated that inhibition of STAT-3 impedes T-cell proliferation and survival, thereby maintaining the function of regulatory T cells (Tregs) [34].

Findings indicate an increased percentage of the Treg population in the colon, along with elevated TGF-β levels, while blocking the COX-2 pathway and upregulating PTEN, an inhibitor of STAT-3 signaling (Figure 5). PTEN’s activation leads to the transcription of pro-inflammatory factors COX-2 and iNOS [35]. iNOS activation in the intestine results in nitric oxide (NO) release, compromising colonic integrity through the formation of peroxynitrite, a potent oxidizing agent [36]. Conversely, increased COX-2 expression during colitis produces PGE2 and PTEN, exacerbating hyperemia and edema. In DSS-induced models, expression levels were restored following the administration of *Bacillus*-loaded nanoparticles [32].

Pro-inflammatory cytokines such as TNF-α and IL-6 decreased when Lactobacillus-enriched colon tissues were analyzed [18,37]. Similarly, oral administration of the *L. plantarum* strain (CAU1055) resulted in a significant reduction in COX-2, iNOS, TNF-α, and IL-6 levels. In this study, the *E. lactis* and HPL combination effectively managed colitis by modulating downstream inflammatory effectors such as COX-2 and iNOS (Figure 3 and Figure 5). The data indicate that *E. lactis* supplementation may enhance antioxidant defenses and manage colitis-related inflammation, necessitating further investigation into its medicinal potential and optimal dosage. The primary limitation of this study is the utilization of a C57BL/6j mouse model, which may not adequately represent the complexities of human colitis and colorectal cancer, along with a restricted sample size that may affect the generalizability of the findings. The brief treatment duration does not adequately assess long-term effects, and the mechanisms through which *E. lactis* and HPL confer benefits were not comprehensively investigated. Furthermore, although we provide evidence of their beneficial effects, we did not compare these treatments with established therapies or evaluate their applicability to human populations. In addition, further research is necessary in order to determine the optimal dosage and administration regimen for *E. lactis* and HPL. Addressing these limitations in future studies can validate our findings and provide a more comprehensive understanding of the therapeutic potential of *E. lactis* and HPL in managing colitis and CRC.

## 4. Materials and Methods

### 4.1. Preparation of Protein Hydrolysate

Hasawi rice was purchased from eastern cultivars in Saudi Arabia. Enzymatic hydrolysates of Hasawi rice were prepared using papain from the extraction solution. Hydrolysis was conducted at 60 °C for five hours, with the pH adjusted to 7.4 [38]. Activated carbon was used to remove phenylalanine from protein hydrolysates [39]. Following this process, the hydrolysate was freeze-dried.

### 4.2. Effect of E. lactis on Pancreatin Tolerance Test

In order to test pancreatin tolerance, 100 µL of bacterial culture in MRS broth was added to 10 mL of MRS broth with 0.5% (*w*/*v*) pancreatin. A control set was also prepared by inoculating the bacterial suspension into pancreatin-free MRS broth. Incubation was carried out for 48 h at 40 °C, and the viable bacterial cell count was determined and expressed as 10^8^ CFU/mL at 24 and 48 h [39].

### 4.3. Safety Assessment

The study evaluated the probiotic potential and assessed the safety of *Lactobacillus mucosae* strains isolated from Donkey’s lactation in vitro [37].

#### 4.3.1. Hemolytic Activity

In order to prepare blood agar plates, 5% mouse blood was added to Brain Heart Infusion (BHI) agar, and a hemolytic test was conducted [40]. The test isolate was placed on freshly made blood agar plates. The plates were then kept at 40 °C for 24 to 48 h to observe α, β, and γ hemolysis patterns.

#### 4.3.2. DNase Activity

Rastogi et al. [22] maintained the test isolate at 40 °C for 72 h without oxygen after spreading it on DNase agar plates (Himedia, Mumbai, India). Colonies were examined for the presence of a clear zone following drenching with 3% HCl for eight minutes [41].

#### 4.3.3. AOM/DSS-Induced Colorectal Cancer Animal Model

Male C57BL/6j mice, aged eight weeks, were obtained from the animal facility unit at King Faisal University’s College of Science. The mice were housed under controlled conditions, with a temperature of 22 ± 2 °C, relative humidity of 55 ± 5%, and a 12 h light/dark cycle. They were provided with ad libitum access to a high-protein–fat diet and water. The mice were randomly divided into five groups (each consisting of 6 mice). These groups included Group-1—an untreated normal control group receiving only PBS treatment, Group-2—an AOM/DSS control group subjected solely to AOM/DSS treatment, Group-3—an *E. lactis* group exposed to AOM/DSS treatment along with *E. lactis* supplementation at a dosage of 1 × 10^8^ CFU/kg/day, Group-4—an *E. lactis*-HPL group receiving AOM/DSS treatment combined with *E. lactis* supplementation (1 × 10^8^ CFU/kg/day) along with HPL at a dosage of 1 mg/kg BW, and Group-5—an HPL group subjected to AOM/DSS treatment with HPL supplementation at a dosage of 1 mg/kg BW.

Colon carcinogenesis was induced in these mice by intraperitoneal injection of 10 mg/kg AOM (Sigma-Aldrich, St. Louis, MO, USA) at the beginning of Week 1, followed by administration of 2.5% DSS (Sigma-Aldrich) in their daily drinking water. Subsequently, the mice were provided with regular drinking water for two weeks to expedite recovery. AOM administration was conducted once in Week 1, whereas DSS administration AOM administration was conducted once in Week 1, whereas DSS administration was performed three times during the experimental period, specifically in Weeks 1–2, 4–5, and 7–8. The body weights of the mice were documented on a weekly basis during the study period. At the conclusion of Week 11, the mice were euthanized, and their colons were excised. Following the measurement of colon lengths, the specimens were washed with PBS and subsequently longitudinally incised. Gross tumors were quantified, and each colon was divided longitudinally into two halves. One-half of the colon was fixed in 10% neutral-buffered formalin (Sigma-Aldrich) and subjected to hematoxylin and eosin (H&E) staining. In contrast, the other half was preserved in liquid nitrogen for subsequent extraction and analysis, including real-time PCR, enzyme-linked immunosorbent assays (ELISA), and Western blot analyses. The institutional animal care and use committee of the Deanship of Scientific Research, King Faisal University (approval number KFU-REC-2023-MAY-ETHICS930) approved all mice procedures. In addition, the study procedures adhered to the Care and Use of Laboratory Animals guidelines.

#### 4.3.4. Disease Activity Index (DAI) Measurement

The severity of colitis was assessed daily using the DAI score throughout the trial. The scoring methodology outlined by Alzahrani et al. [42] was utilized to calculate the DAI. This composite index included factors such as the presence of blood in stool, stool consistency, and weight loss. Scores were assigned based on predefined criteria for each factor and summed accordingly. Specifically, a score of 0 was assigned for 0% weight loss, while weight losses of 0–10%, 11–15%, 16–20%, and >20% received scores of 1, 2, 3, and 4, respectively. Stool consistency was evaluated, with normal stools receiving 0 points, mildly watery stools receiving 2 points, and diarrhea receiving 4 points. Hematochezia was scored as 0 in its absence and 4 in its presence. Weights were recorded prior to the experiment and consistently at the same time each day during the study to minimize measurement errors.

#### 4.3.5. Analysis of Aberrant Crypt Foci (ACF)

Kowalczyk et al. [43] developed a technique to analyze colonic aberrant crypt foci (ACF). Colons were extracted, sectioned longitudinally, and cleaned using a saline solution. Each colon was segmented into three equal-length sections (proximal, middle, and distal), flattened between filter papers, and preserved in 10% buffered formalin. Following the application of a 0.2% methylene blue solution, ACF were visualized using a light microscope, and their locations were documented. The area of each colon slice was measured utilizing NIS-Elements microscope imaging software v4.x. Counts of ACF and aberrant crypts (AC) were assessed for each specimen.

#### 4.3.6. Histopathological Analysis

Tissues from distal colon sections were fixed in 10% formaldehyde, embedded in paraffin blocks, and underwent H&E staining for histopathological examination [32]. Pathologists evaluated histological alterations in randomly selected observation fields based on the severity of colitis using an optical microscope (Eclipse 80i, Nikon Inc., Melville, NY, USA) at a magnification of approximately 40×.

#### 4.3.7. Analysis of ELISA

Following centrifugation at 3000× *g* for 15 min at 4 °C, serum samples were stored at −80 °C. Levels of interleukin (IL)-6, tumor necrosis factor (TNF)-α, and IL-1β were quantified using an enzyme-linked immunosorbent assay (ELISA, PeproTech, Jersey City, NJ, USA).

#### 4.3.8. Analysis of Protein Expression

Colonic tissues were homogenized in radioimmunoprecipitation assay (RIPA) lysis buffer containing a protease inhibitor mixture. Protein expression was measured by subjecting 20 μg of homogenized protein to 4–12% sodium dodecyl sulfate–polyacrylamide gel electrophoresis (SDS-PAGE), then transferred to a PVDF membrane. Primary polyclonal rabbit antibodies targeting PCNA, PTEN, VEGF, COX2, and STAT-3 (dilution 1:1000, Biorybt, UK) were applied to the membranes. Signal quantification was performed using the LICOR chemiluminescent detector (Licor, Lincoln, CA, USA).

#### 4.3.9. Statistical Analysis

The statistical analysis was conducted using the software application GraphPad (Version 8.0). A unidirectional analysis of variance was employed to compare the results, with the threshold for the observed disparities set at a significance level of *p* < 0.05.

## 5. Conclusions

This study demonstrated that *Enterococcus lactis* effectively alleviates colitis induced by dextran sulfate sodium in mice, thereby improving DAI scores, gut permeability, and SOD. The combination of *E. lactis* with HPL further enhances these benefits by significantly reducing pro-inflammatory markers and cell death-related proteins. The findings indicate the potential role of probiotics in colitis management and suggest that HPL may improve the efficacy of *E. lactis*. Probiotics demonstrate potential in the treatment of colitis-associated colorectal cancer. However, additional clinical trials are required to verify their safety and efficacy.

## Data Availability

The original contributions presented in the study are included in the article, further inquiries can be directed to the corresponding author.

## Abbreviations

AOM	Azoxymethane
DSS	Dextran Sulfate Sodium
*E. lactis*	*Lactobacillus lactis*
CFU	Colony-forming Unit
HPL	Hydroxyethyl Starch–Lanthanum Complex
FITC-dextran	Fluorescein isothiocyanate-dextran
SOD	Superoxide Dismutase
U/mg protein	Units per milligram of protein
